# Association Between COVID-19 and Neurological Diseases: Evidence from Large-Scale Mendelian Randomization Analysis and Single-Cell RNA Sequencing Analysis

**DOI:** 10.1007/s12035-024-03975-2

**Published:** 2024-02-01

**Authors:** Lin Huang, Yongheng Wang, Yijie He, Dongyu Huang, Tong Wen, Zhijie Han

**Affiliations:** 1 Department of Bioinformatics, School of Basic Medicine, Chongqing Medical University, Chongqing, China; 2 International Research Laboratory of Reproduction & Development, Chongqing Medical University, Chongqing, China

**Keywords:** COVID-19, Neurological diseases, Mendelian randomization, Single-cell RNA sequencing analysis

## Abstract

Observational studies have suggested that SARS-CoV-2 infection increases the risk of neurological diseases, but it remains unclear whether the association is causal. The present study aims to evaluate the causal relationships between SARS-CoV-2 infections and neurological diseases and analyzes the potential routes of SARS-CoV-2 entry at the cellular level. We performed Mendelian randomization (MR) analysis with CAUSE method to investigate causal relationship of SARS-CoV-2 infections with neurological diseases. Then, we conducted single-cell RNA sequencing (scRNA-seq) analysis to obtain evidence of potential neuroinvasion routes by measuring SARS-CoV-2 receptor expression in specific cell subtypes. Fast gene set enrichment analysis (fGSEA) was further performed to assess the pathogenesis of related diseases. The results showed that the COVID-19 is causally associated with manic (delta_elpd, − 0.1300, *Z*-score: − 2.4; *P* = 0.0082) and epilepsy (delta_elpd: − 2.20, *Z*-score: − 1.80; *P* = 0.038). However, no significant effects were observed for COVID-19 on other traits. Moreover, there are 23 cell subtypes identified through the scRNA-seq transcriptomics data of epilepsy, and SARS-CoV-2 receptor TTYH2 was found to be specifically expressed in oligodendrocyte and astrocyte cell subtypes. Furthermore, fGSEA analysis showed that the cell subtypes with receptor-specific expression was related to methylation of lysine 27 on histone H3 (H3K27ME3), neuronal system, aging brain, neurogenesis, and neuron projection. In summary, this study shows causal links between SARS-CoV-2 infections and neurological disorders such as epilepsy and manic, supported by MR and scRNA-seq analysis. These results should be considered in further studies and public health measures on COVID-19 and neurological diseases.

**Supplementary Information:**

The online version contains supplementary material available at 10.1007/s12035-024-03975-2.

## Introduction

Coronavirus disease 2019 (COVID-19), caused by severe acute respiratory syndrome coronavirus 2 (SARS-CoV-2), has emerged worldwide, resulting in 600 million infections and over 6 million deaths as of August 2022 according to the World Health Organization (WHO) report [1]. While it was initially thought to be restricted to the respiratory system, the adverse effects of COVID-19 on the central and peripheral nervous systems have been increasingly recognized [2].

Although the pathogenic mechanism and epidemiological features of SARS-CoV-2 are still largely unclear, some observational studies have reported findings suggesting that COVID-19 may be associated with an increased risk of neurological diseases. These neurologic disorders could be considered in three categories: (1) related to central nervous system, including headache [3, 4], confusion or impaired consciousness [5, 6], meningoencephalitis [7, 8], cerebrovascular disease [9, 10], and stroke [11, 12]; (2) related to peripheral nervous system, including taste and smell impairment [13, 14] and vision impairment [15, 16]; (3) muscular injury [17, 18]; and (4) related to altered mental status, including anxiety disorder [19] and psychotic disorder [20]. COVID-19 neuropathogenesis might involve the invasion of the nervous system by SARS-CoV-2 and cause neurological complications. Previous studies have shown that SARS-CoV-2 virus particles were found in the frontal lobes and cerebrospinal fluid (CSF) of patients with COVID-19 [21, 22]. Moreover, SARS-CoV-2 replication could be detected in the brains of human angiotensin-converting enzyme 2 (hACE2) knock-in mice and human neural tissue [23, 24]. Additionally, SARS-CoV-2 belongs to the same Betacoronavirus (β-CoV) genus as SARS-CoV, and they share approximately 76% amino acid identity [25–27]. Most β-CoVs exhibit a propensity for neuroinvasion [28]. However, the claims of neuroinvasion by SARS-CoV-2 in literatures have thus far been inconsistent [23, 29–31].

Furthermore, SARS-CoV-2 enters host cells using the receptor angiotensin-converting enzyme 2 (ACE2) and transmembrane serine protease 2 (TMPRSS2) as same as SARS-CoV [32], but they alone cannot explain the significant differences in the primary infection sites and clinical manifestations exhibited by SARS-CoV-2 and SARS-CoV, suggesting the involvement of other receptors in SARS-CoV-2 host interactions [33, 34]. Therefore, a comprehensive investigation of the relationship between SARS-CoV-2 infection and neurological diseases, along with an exploration of the receptors that have played a role in the virally infected brain, is needed.

Mendelian randomization (MR) is an analytic approach that uses genetic variation as the proxy to examine the potential causal effect of an exposure (e.g., COVID-19) on the outcome (e.g., Neurological disorders). Different from observational studies, confounding bias can be minimized in MR studies because genetic variants are randomly allocated to the individual at birth. Similarly, reverse causation can be avoided because genetic variants are allocated before the development of the disease [35, 36]. In this study, we sought to evaluate the associations of COVID-19 with the risk of neurologic disorders using Causal Analysis Using Summary Effect estimates (CAUSE) analysis, a novel MR method that can avoid more false positives caused by correlated horizontal pleiotropy [37]. Moreover, single-cell RNA sequencing (scRNA-seq) analysis and fast gene set enrichment analysis (fGSEA) were employed to characterize and compare distinct cell subtypes between conditions and explore disease-related pathways [38].

## Materials and Methods

### Overview

**Fig. 1:**
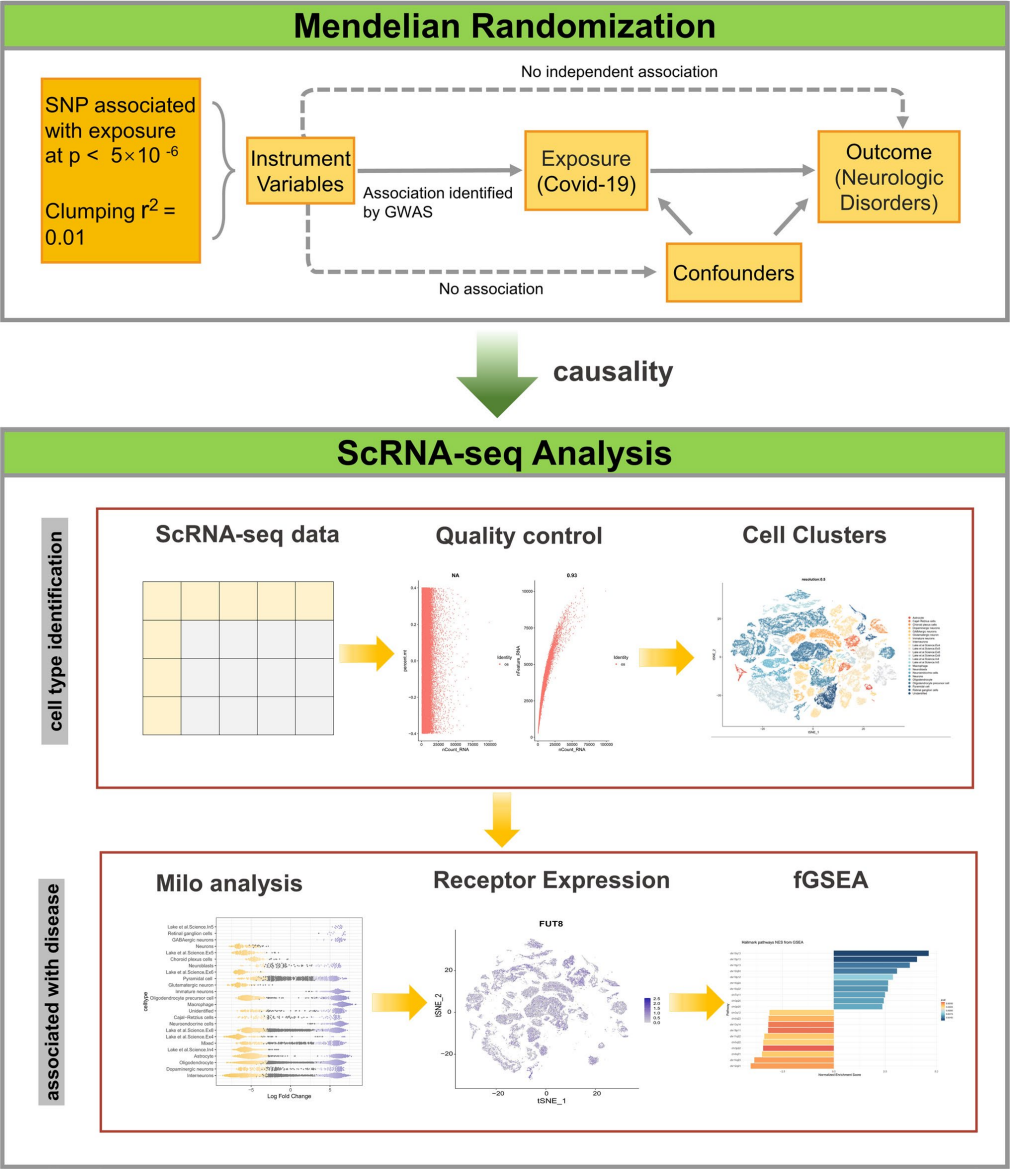
Outline of the analyses performed. Using multiple data sources, this study performed Mendelian randomization (MR) analysis to investigate the causal relationship of SARS-Cov-2 infections with neurological diseases. Significant MR associations were investigated further with single-cell RNA sequencing (scRNA-seq) analysis

MR is an increasingly popular approach that estimates the causal effect in observational epidemiological studies. Traditional MR methods rely on several strong, untestable assumptions, including (i) the genetic variant must be truly associated with the exposure; (ii) the genetic variant should not be associated with confounders of the exposure-outcome relationships; (iii) the genetic variant should only be related to the outcome of interest through the exposure under study. However, some of the strong assumptions required to make causal inferences in MR described previously are often violated, and thus, biases are introduced. It is thoughtful to take into account whether the interpretation of results violates the assumptions of this approach. Nevertheless, more robust methods have been proposed to overcome these limitations and enhance the discrimination of genetic associations with the outcome, distinguishing whether it is caused by a genetic causal relationship or by correlated and uncorrelated pleiotropic effects. Here, we performed a new MR method, CAUSE, which accounts for correlated and uncorrelated horizontal pleiotropic effects to assess the causal relationships of SARS-CoV-2 infections with neurological diseases. Then, we further conducted a single-cell transcriptome analysis to measure the expression and distribution of SARS-CoV-2 receptors and fGSEA, aiming to elucidate potential disease mechanism. The corresponding flow chart is presented in Fig. 1.

### GWAS Summary Statistics for Exposure and Outcomes

We use CAUSE to test for causal effects of COVID-19 on 176 neurological disorders according to the 11th revision of International Classification of Diseases (ICD-11) [39]. The Genome-Wide Association Study (GWAS) summary statistics for exposure and outcomes are available in the IEU OpenGWAS database [40], and the detailed information is shown in Supplementary Table S1. We obtained the GWAS summary statistics according to the following selection criteria: (i) We identified neurological disorders within the chapters of Neurology in ICD-11, encompassing the following three chapters: “06 Mental, behavioral, or neurodevelopmental disorders,” “07 Sleep–wake disorders,” and “08 Diseases of the nervous system.” It is noteworthy that ICD-11 defines the concept of “multiple parenting,” allowing a disease to be classified in several chapters. For example, “Stroke” is list in several chapters: “8B20 Stroke not known if ischaemic or haemorrhagic,” “24 Factors influencing health status or contact with health services.” We still include it in our study. As long as the disease is in the neurological sections, it is included in the analysis; (ii) only the European participants were included. (iii) We prioritized studies with the largest possible sample sizes and the highest number of single-nucleotide polymorphisms (SNPs), and subsequently take into account the closest possible publication dates. Finally, a total of 173 GWAS datasets were selected for the MR analysis which include one COVID-19 study as the exposure group and 172 neurological disorder studies as the outcome groups.

### IV Identification and MR Analysis

CAUSE computes joint density of all summary statistics for each variant, To account for this, this approach includes as much information from all variants as possible, including weakly associated variants (*P* < 10^–3^) and those with low mutual LD (*r*^2^ > 0.01), aiming to improve power [37]. Therefore, SNPs associated with COVID-19 at a slightly more lenient significance threshold (*P* < 5 × 10^−6^) were selected as potential instrumental variables (IVs). Then, based on the European-based 1000 Genomes projects reference panel, the linkage disequilibrium (LD) threshold for clumping was set to *r*^2^ > 0.01 within a 10,000 kb window using the “ld_clump” function of the R package “ieugwasr” (https://mrcieu.github.io/ieugwasr/). Finally, we estimated the nuisance parameters using the “est_cause_params” function of the R package “cause” (https://github.com/jean997/cause). Based on these nuisance parameters, the expected log pointwise posterior density (ELPD) was computed for sharing, causal, and null model, respectively.

### ScRNA-seq Data Sources and SARS-CoV-2 Receptor Selection

To further validate the results of the MR analysis and explore the infection pathway of SARS-CoV-2 in these disorders, we selected neurological disorders with causal relationship with SARS-CoV-2 infection for scRNA-seq analysis. Among these identified disorders, only the scRNA-seq raw data for epilepsy are available, obtained from the Pfisterer U et al. [41]. Therefore, the subsequent analysis focuses only on the mechanisms of epilepsy. Furthermore, due to that the specific receptors for viruses are key to promote them entry into host cells and pathogenesis [32–34], we measured the expression of SARS-CoV-2 receptors in various cell subtypes and different disease states. The SARS-CoV-2 receptors were selected by searching all the possible studies in the databases of PubMed (http://www.ncbi.nlm.nih.gov/pubmed) and Google Scholar (http://scholar.google.com/) using the keywords: “SARS-CoV-2,” “COVID-19,” and “receptors”.

### ScRNA-seq Analysis

The whole process of scRNA-seq data analysis was performed by R package Seurat [42]. Particularly, the low-quality cells with less than 500 detected genes or higher than 100,000 or mitochondrial gene expression exceeded 20% were excluded from the following analysis. SCTransform was used for normalization, variance stabilization, and feature selection. All data were scaled to weight for downstream analysis, and principal component analysis (PCA) and T-distributed Stochastic Neighbor Embedding (t-SNE) dimension reduction with top 40 principal components (PCs) were performed. A nearest-neighbor graph was calculated using FindNeighbors, followed by clustering identifying FindClusters with a resolution of 0.5. Marker genes of each cluster were identified by the FindAllMarkers with Wilcox *t* test, and annotation was performed based on databases including CellMarker and PanglaoDB [43, 44]. Finally, 106,527 cells were clustered. After cell type identification, feature plots were visualized to identify the expression of 20 receptors in each cluster.

### Differential Abundance Test and Receptor Cellular Distribution

To further explain the causal association between SARS-CoV-2 infection and risk of epilepsy, we performed a differential abundance testing algorithm to identify disease-specific cell populations and explored the potential neuroinvasion routes by measuring SARS-CoV-2 receptor expression levels in each cell type. the differential abundance analysis was analyzed via the R package “Milo” by allocating cells to partially overlapping neighborhoods on a k-nearest neighbor (KNN) graph [42]. With calculated log2 fold change and FDR, clusters having statistically significant differential abundance were determined. We create a “Milo object” from a Seurat object that includes PCA and TSNE dimensionality reduction. Then, the “makeNhoods” function was employed to define representative neighborhoods, with the parameters “prop,” “k,” and “d” all set to their default values. The distribution of neighborhoods size is shown in Supplementary Figure S1. Conducting differential abundance analysis between conditions (normal vs epilepsy) in all neighborhoods, the procedure applied a weighted false discovery rate (FDR) to correct for multiple hypotheses. Subsequently, we measured the cellular distribution of SARS-CoV-2 receptors and compared the expression of each receptor among the cell subtypes using the “FeaturePlot” function of the R package “Seurat” [39].

### Fast Gene Set Enrichment Analysis

To investigate and explore the biological signaling pathway, fGSEA was utilized respectively for oligodendrocyte and astrocyte cell subtypes in comparison with the remaining cell subtypes. The top 10 terms from C2 curated gene set and C5 ontology gene sets were presented. Significantly enriched pathways were illustrated based on NES (Net enrichment score) and *P* value. Gene sets with |NES|> 1and FDR *q* < 0.05 were deemed to be significantly enriched.

## Results

### Identification of Cell Subtypes

**Fig. 2:**
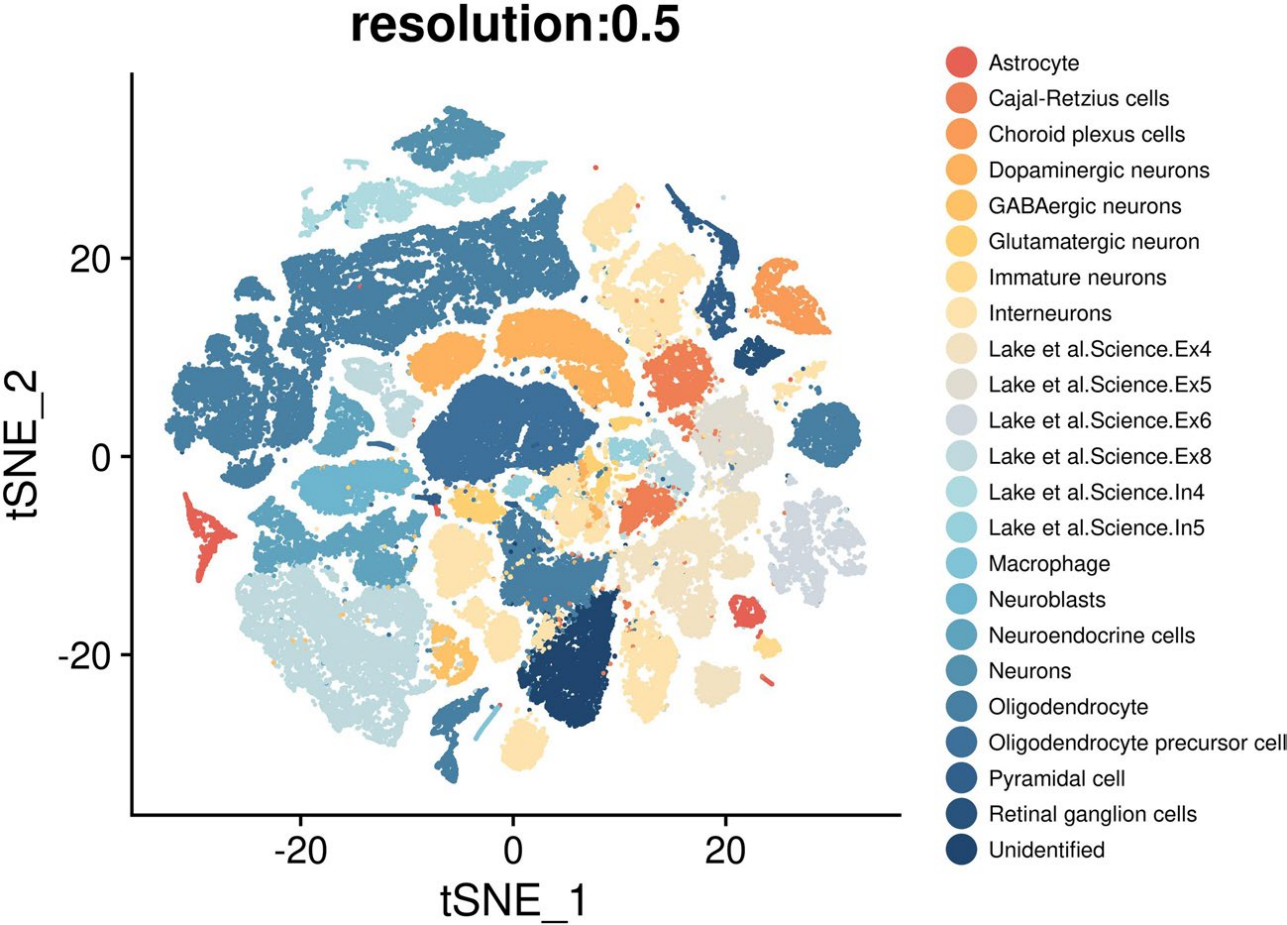
T-distributed stochastic neighbor embedding (t-SNE) plot of samples revealing the different clusters

We conducted the scRNA-seq transcriptomics analysis on temporal cortex of 19 samples (including nine epilepsy patients and 10 controls without any neurological disorders) provided by Pfisterer U et al*.* [41]. After quality control and data normalization, a total of 29,212 RNA features and 106,527 cells were retained for downstream analysis. T-SNE was used for dimensional reduction for high-quality cells, and 23 different clusters were obtained eventually (Fig. 2).

### Causal Effects of COVID-19 on Manic

For the manic trait, genetic predisposition to COVID-19 is associated with the risk of it. As Table 1 shows, when compared with the sharing model, the estimated difference in expected log pointwise posterior density (delta EPLD) in the causal models is negative, and significant differences are presented (delta EPLD =  − 0.1300). Furthermore, the fitted delta EPLD of the causal model is still significantly better than the sharing model (*z*-score =  − 2.4 and *p* value = 0.0082). This revealed different posterior distributions and proportions of correlated pleiotropic SNPs in the two models.

**Table 1:** The results of causality of SARS-Cov-2 infection and manic by comparing causal and sharing model

Model 1	Model 2	Δ ELPD	s.e. Δ ELPD	*Z*-score	*p* value
Null	Sharing	− 0.0018	0.0015	− 1.2	0.1100
Null	Causal	− 0.1300	0.0560	− 2.4	0.0088
Sharing	Causal	− 0.1300	0.0550	− 2.4	0.0082

Model 1 and Model 2 imply the models being compared. When the estimated difference in ELPD (Δ ELPD) = ELPDC − ELPDS is negative, model 2 is a better fit. s.e. ΔELPD estimated standard error of ΔELPD; *Z*-score = ΔELPD s.e. Δ ELPD

### Causal Effects of COVID-19 on Epilepsy

For the epilepsy trait, there is a causal association between COVID-19 and it, suggesting that COVID-19 infection may increase the risk of epilepsy. As Table 2 shows, the fit of both the shared and causal models was significantly different from the null model (no causal or shared effects). The causal model trends to have a better fit than the sharing model and is statistically better than it.

**Table 2:** The results of causality of SARS-Cov-2 infection and epilepsy by comparing causal and sharing model

Model 1	Model 2	Δ ELPD	s.e. ΔELPD	*Z*-score	*p* value
Null	Sharing	− 0.11	0.12	− 0.86	0.190
Null	Causal	− 2.30	1.40	− 1.70	0.045
Sharing	Causal	− 2.20	1.20	− 1.80	0.038

### Cell Subtypes Associated with Epilepsy

**Fig. 3:**
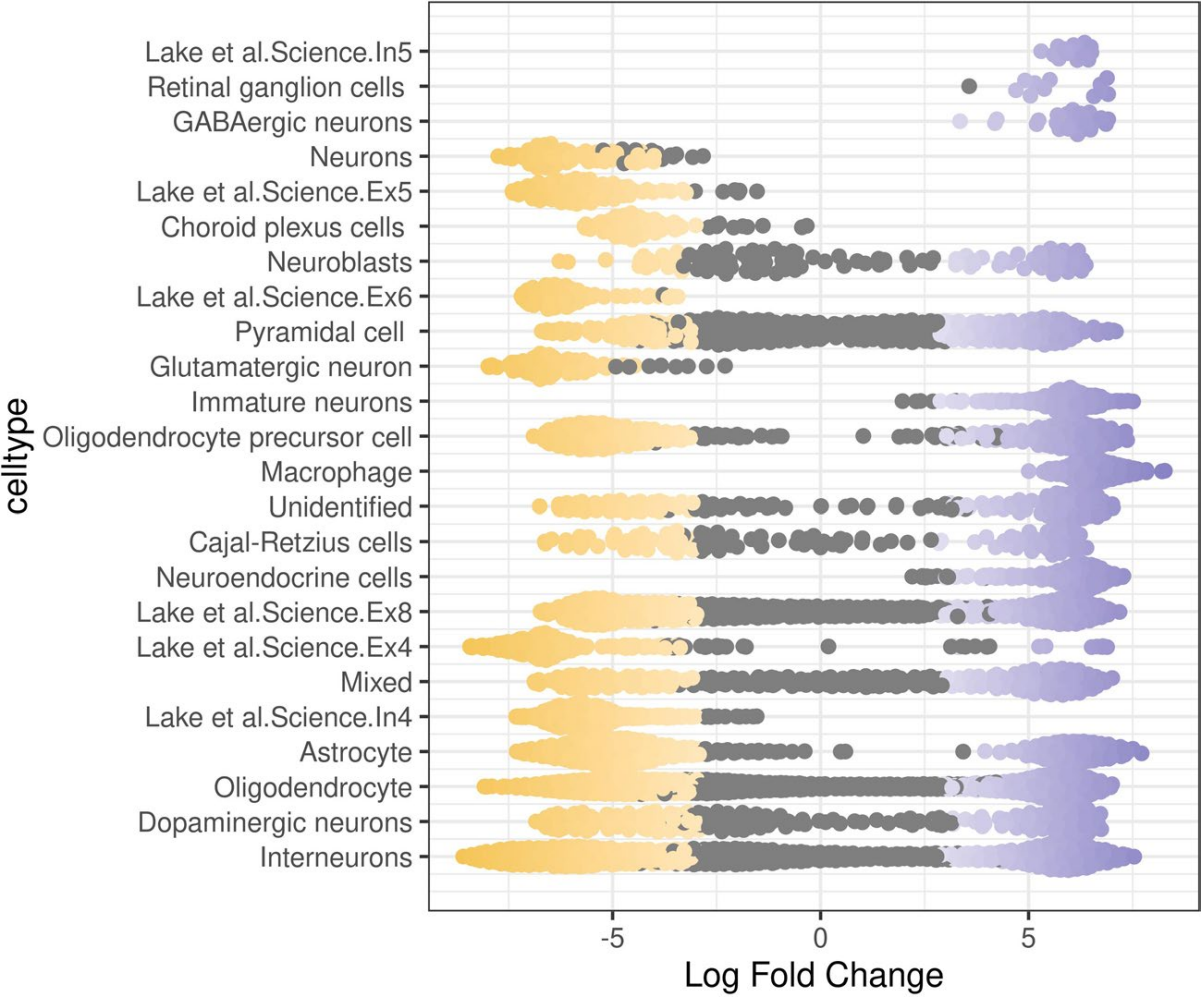
Beeswarm plot of the distribution of log fold change in abundance between conditions in neighborhoods from different cell type clusters. Differential abundance neighborhoods at FDR 10% are colored (red = down, blue = up). Cell subtypes detected as differential abundance through clustering are annotated in the left sidebar

Next, we utilized Milo to calculate cell-neighbourhoods and performed differential cell abundance testing between control and epilepsy confirming that the abundance of cellular states was substantially different in all clusters (Fig. 3; spatial false discovery rate (FDR) < 0.05). The plot shows comparisons along the control and epilepsy, with neighborhoods that are significantly differentially abundant epilepsy samples shown in purple and neighborhoods that are significantly differentially abundant control samples shown in yellow. We identified that pyramidal, oligodendrocyte, mixed, Ex8, interneuron, and dopaminergic are significantly enriched both in epilepsy and healthy subjects and continuously distributed between them. This suggests that these cell subtypes are present throughout the duration of epilepsy. Then, fGSEA was performed to identify the functional enrichment of cell subtypes with receptor-specific expression (Supplementary Figure S2 to S5). Enrichment term exhibited that was mainly astrocyte cell subtypes associated with H3K27ME3, neuronal system, aging brain, neurogenesis, neuron projection, and other neurological enrichment. However, there was no significant neurological enrichment in selected terms for oligodendrocyte cell subtypes.

### SARS-CoV-2 Receptor Expression

**Fig. 4:**
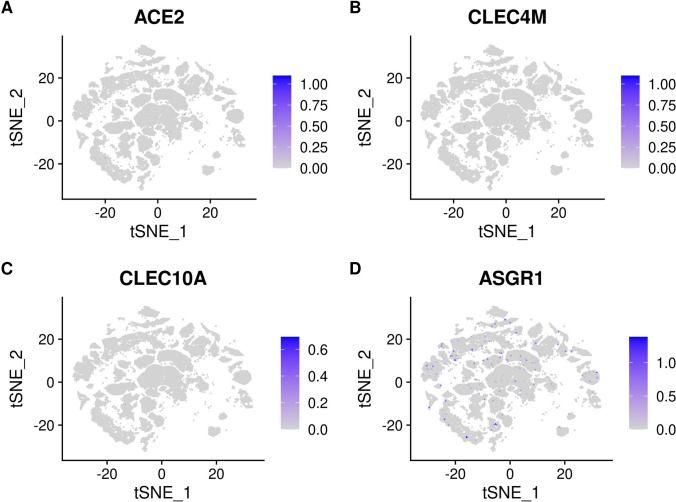
**A** Expression of ACE2 in different clusters of cells. **B** Expression of CLEC4M in different clusters of cells. **C** Expression of CLEC10A in different clusters of cells. **D** Expression of ASGR1 in different clusters of cells

**Fig. 5:**
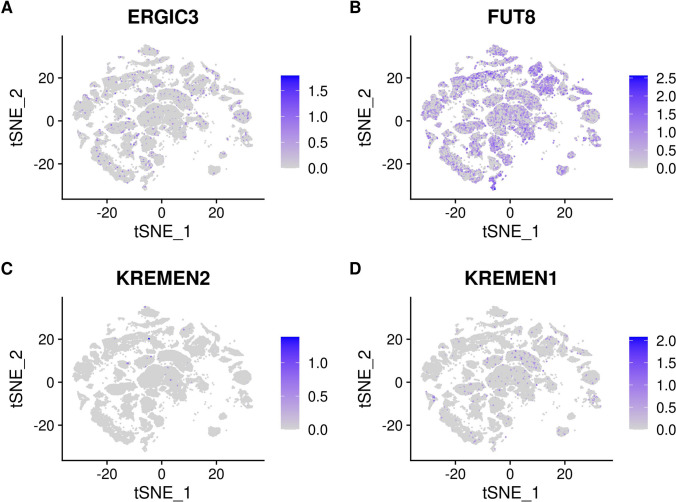
**A** Expression of ERGIC3 in different clusters of cells. **B** Expression of FUT8 in different clusters of cells. **C** Expression of KREMEN2 in different clusters of cells. **D** Expression of KREMEN1 in different clusters of cells

**Fig. 6:**
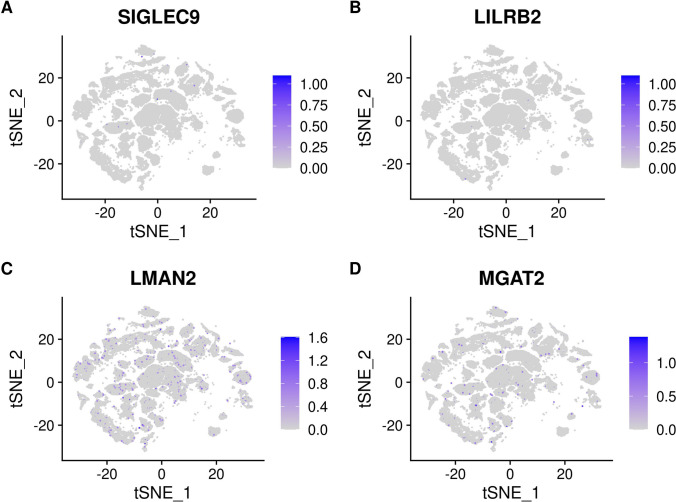
**A** Expression of SIGLEC9 in different clusters of cells. **B** Expression of LILRB2 in different clusters of cells. **C** Expression of LMAN2 in different clusters of cells. **D** Expression of MGAT2 in different clusters of cells

**Fig. 7:**
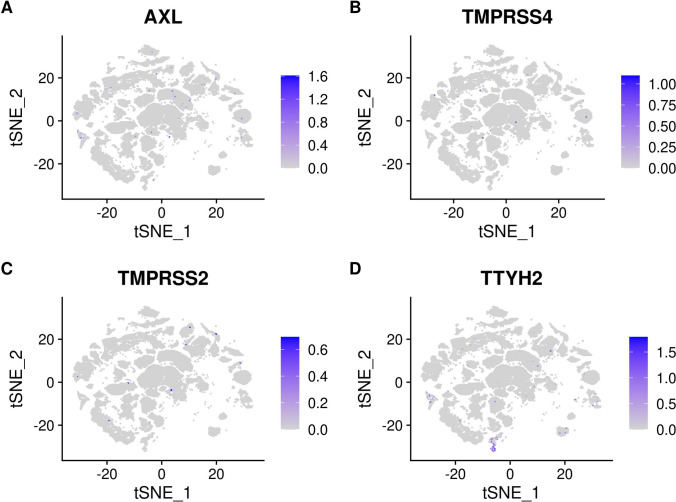
**A** Expression of AXL in different clusters of cells. **B** Expression of TMPRSS4 in different clusters of cells. **C** Expression of TMPRSS2 in different clusters of cells. **D** Expression of TTYH2 in different clusters of cells

The cell-specific expression and distribution of the receptors determine the tropism of virus infection, which has a major implication for understanding its pathogenesis and designing therapeutic strategies. Our results showed that TTYH2 is specifically highly expressed in oligodendrocyte and astrocyte cell subtypes. The ACE2 and TMPRSS2 expressing cell ratio is relatively low, which is consistent with previous studies [45]. Expression of the other five receptors is also low. In addition, the expressions of ERGIC3, ASGR1, FUT8, KREMEN1, and LMAN2 are relatively high and not specifically expressed in any cluster. The expressions of receptors in each cluster are demonstrated in Figs. 4, 5, 6, and 7. Considering that oligodendrocyte and astrocyte cells have also been confirmed to play a key role in epilepsy pathogenesis [46, 47], the causal link between SARS-Cov-2 infection and epilepsy may be associated with these two cell subtypes.

## Discussion

In this study, we performed MR analyses that found genetic liability to COVID‐19 to be associated with increased risk of epilepsy or manic among the 176 neurological disorder outcomes tested, which is consistent with multiple epidemiological studies reporting an association between neurological disorders and COVID-19 disease. We also performed the scRNA-seq analyses and fGSEA to further explain potential infection mechanisms.

Previous clinical studies have suggested that COVID-19 may trigger clinical manifestations of neurological disorders, notably manic and epilepsy. These complications have been frequently reported in association with COVID-19 [48, 49]. Approximately 13 of 1744 patients with COVID-19 (0.7%) have been reported to have steroid-induced mania and psychosis [50]. Another study firstly reported that specific IgG antibody for SARS-CoV-2 is detected from a specimen of CSF in a COVID-19 patient with manic-like symptoms [51]. Similarly, SARS-CoV-2 was detected in the serum and CSF of SARS patients with persistent epilepsy [52]. The MR results consistently indicated an elevated risk of genetic predisposition to manic and epilepsy in connection with COVID-19, aligning with epidemiological observations. In the single-cell analysis of epilepsy, the expression of receptors related to COVID-19 can be categorized into three scenarios: receptors with low expression level, such as ACE and CLEC4M; receptors exhibiting high expression across all cell clusters, such as FUT8 and KREMEN1; and the last category comprises receptors with specific high expression, exemplified by TTYH. Tweety homologs (TTYHs) include three members (TTYH 1–3) in humans that have been implicated in the pathogenesis of epilepsy. TTYH2 not only acts as a receptor for the SARS-CoV-2 but also as a calcium-activated chloride channel (CaCCs). Voltage-gated chloride channels (ClC) participate in the epileptogenesis process of epilepsy, and the inhibition of ClC may have anti-epileptic effect [53, 54]. Thus, the unbalanced expression levels of TTYH2 were one of the reasons for concomitant epilepsy in patients with COVID-19. The associations between COVID-19 and epilepsy and manic emphasize incorporating early intervention protocols into the care of COVID-19 patients which could contribute to better outcomes and improved quality of life.

Furthermore, mechanisms that increase the risk of neurological disorders in patients with COVID‐19 are complex. The fGSEA is a powerful method that focuses on gene sets to identify shared common biological functions or regulations. Epilepsy is a disease of aging; the incidence of epilepsy is highest in people over 65 [55]. H3K27me3 is a highly conserved repressive histone modification found in many central nervous system diseases and implicated in various neuronal processes [56, 57]. Previously, studies indicate that H3K27me3 levels increase with age in neurons, and high levels of H3K27me3 are potentially important in preserving adult neural function [58–60]. This suggests the potential relevance of this modification in various neuronal processes. Our findings also support the hypothesis that SARS-CoV-2 can enter the nervous system, thereby triggering the onset and progression of neurological diseases.

Apart from manic and epilepsy, no associations were found with COVID-19 exposures. The possible explanation is that if COVID-19 has a causal role in any of the outcomes’ susceptibility or severity, their effects may have been too small to be detected with our current sample sizes. It is noteworthy that relevant risk factors can still have clinical utility in identifying patients at risk, even if causality is disproved.

This study employed the CAUSE and single-cell transcriptome analysis to comprehensively assess the association between COVID-19 infection and prevalent neurological diseases. Additionally, fGSEA was conducted to evaluate the pathways implicated in the pathogenesis of epilepsy. However, our study also has limitations. Firstly, there are several modeling assumptions to be made when using MR, specifically that the genetic variants do not affect the considered outcomes through pathways independent of the exposure. Although this can never be completely ruled out, well-powered randomized trials are needed to conclusively assess this association. Secondly, due to the lack of scRNA-Seq data from patients infected with COVID-19 and causing neurological disease, we were unable to analyze receptor expression therein. Thirdly, both GWAS and scRNA-Seq data used in this study were predominantly obtained from European population ancestry; thus, these findings should be interpreted with caution when generalizing to other populations.

## Conclusions

The present MR study unveiled an association between COVID-19 and epilepsy or manic symptoms. Through the analysis of scRNA-seq data, we observed elevated expression of SARS-CoV-2 receptors, particularly TTYH2, in seizure-related cell subtypes. Understanding the interaction of SARS-CoV-2 with these receptors could open avenues for early intervention and treatment, specifically targeting certain brain cell types in neurological diseases. A better understanding of SARS-CoV-2 infection and its interaction with neurological receptors can lead to the exploration of earlier interventions and treatments, focusing on specific brain cell types for the treatment of neurological diseases. The fGSEA results suggest that H3K27me3 may influence the onset of epilepsy, providing a new pathway for researchers to investigate the biological mechanisms of seizures and delve into new therapeutic targets. Overall, our study indicated the potential associations among SARS-Cov-2 infection and epilepsy or manic at genetic and transcriptomic level. Future research is necessary to investigate the underlying mechanisms linking COVID-19 with epilepsy, manic or other neurological diseases.

### Supplementary Information

Below is the link to the electronic supplementary material.

Supplementary file1 (DOCX 85 KB)

## Data Availability

All the data used in this study can be acquired from the original genome-wide association studies and scRNA-seq that are mentioned in the text. Any other data generated in the analysis process can be requested from the corresponding author.
