# Supplementary material for: Association Between COVID-19 and Neurological Diseases: Evidence from Large-Scale Mendelian Randomization Analysis and Single-Cell RNA Sequencing Analysis
Source: Mol Neurobiol. 2024 Feb 1;61(9):6354–65. doi: 10.1007/s12035-024-03975-2 (PMC11339101; doi:10.1007/s12035-024-03975-2)
Supplement: Supplementary file 1 — Supplementary file1 (DOCX 85 KB) [file 12035_2024_3975_MOESM1_ESM.docx]

**Table S1** Demographic characteristics of included GWASs used in the present MR analysis

| **GWAS Dataset ID** | **Tait** | **ICD-11 codes** | **Number of SNPs** | **Sample size (case/control)** |
| --- | --- | --- | --- | --- |
| ieu-b-2 | Alzheimer's disease | 8A20 Alzheimer disease | 10528610 | 21982/41944 |
| finn-b-G6_BELLPA | Bell's palsy | 8B88 Disorders of facial nerve | 16380413 | 1740/195047 |
| finn-b-F5_HISPER | Histrionic personality disorder | 6D10 Personality disorder | 16380428 | 96/212179 |
| finn-b-KRA_PSY_PUERPER | Mental and behabioural disorders of puerperum, not classified elsewhere | 6E2Z Mental or behavioural disorders associated with pregnancy, childbirth or the puerperium, unspecified | 16379784 | 189/123390 |
| ieu-b-43 | frontotemporal dementia (TDP subtype) | 6D83 Frontotemporal dementia | 494577 | 515/2509 |
| finn-b-F5_DEMENTIA_INCLAVO | Dementia, including avohilmo | Dementia | 16380465 | 7395/211397 |
| finn-b-PD_DEMENTIA_EXMORE | Dementia due to Parkinsons disease (more controls excluded) | 6D85 Dementia due to diseases classified elsewhere | 16379396 | 267/111621 |
| finn-b-KRA_PSY_DEMENTIA | Any dementia | Dementia | 16380466 | 5933/212859 |
| finn-b-F5_VASCDEM | Vascular dementia (F5_VASCDEM) | 6D81 Dementia due to cerebrovascular disease | 16380457 | 881/211508 |
| finn-b-R18_CONVU_NOT_ELSEW_CLASSIFIED | Convulsions, not elsewhere classified | 8A68 Types of seizures | 16380331 | 4660/172999 |
| finn-b-KRA_PSY_TIC | Tic disorders (KRA_PSY_TIC) | 8A05 Tic disorders | 16380466 | 172/218620 |
| finn-b-F5_PTSD | Post-traumatic stress disorder | 6B40 Post traumatic stress disorder | 16380382 | 1103/198110 |
| ieu-b-10 | focal epilepsy, all documented cases | 8A6Y Other specified epilepsy or seizures | 4862782 | 9671/29677 |
| finn-b-GE | Generalized epilepsy | 8A61 Genetic or presumed genetic syndromes primarily expressed as epilepsy | 16380451 | 1781/212532 |
| ieu-b-18 | multiple sclerosis | 8A40 Multiple sclerosis | 6304359 | 47429/68374 |
| finn-b-G6_ATYFAC | Atypical facial pain | 8B82 Disorders of trigeminal nerve | 16380408 | 701/195047 |
| finn-b-F5_DISSOCIATIVE | Dissociative [conversion] disorders | 6B60 Dissociative neurological symptom disorder | 16380387 | 662/198110 |
| finn-b-F5_SCHIZOAFF | Schizoaffective disorder | 6A21 Schizoaffective disorder | 16380439 | 2288/208674 |
| finn-b-F5_SCHIZOTYP | Schizotypal disorder | 6A22 Schizotypal disorder | 16380427 | 668/208674 |
| finn-b-F5_SCHIZPER | Schizoid personality disorder | 6D10 Personality disorder | 16380429 | 424/212179 |
| finn-b-G6_GUILBAR | Guillain-Barre syndrome | 8C01 Inflammatory polyneuropathy | 16380463 | 213/215718 |
| finn-b-G6_MERALGIA | Meralgia paraesthetica | 8C11 Mononeuropathies of lower limb | 16380406 | 387/195047 |
| finn-b-F5_PERVASIVE | Pervasive developmental disorders excl. Autism + Asperger | 6A02.Z Autism spectrum disorder, unspecified | 16380457 | 164/216829 |
| finn-b-G6_HORNER | Horner syndrome | 8D8A Focal or segmental autonomic disorders | 16380466 | 159/218530 |
| finn-b-G6_MYOTDIS | Mytonic disorders | 8C71 Myotonic disorders | NA | 95/217056 |
| ebi-a-GCST005647 | Amyotrophic lateral sclerosis | 8B60 Motor neuron disease | 39630630 | 20806/59804 |
| finn-b-KRA_PSY_ANXIETY | Anxiety disorders | 6B0Z Anxiety or fear-related disorders, unspecified | 16380466 | 20992/197800 |
| finn-b-F5_PANIC | Panic disorder | 6B01 Panic disorder | 16380395 | 2376/198110 |
| finn-b-KRA_PSY_MENTALRET | Mental retardation (KRA_PSY_MENTALRET) | 6A00 Disorders of intellectual development | 16380466 | 704/218088 |
| ieu-b-42 | schizophrenia | 6A20 Schizophrenia | 15358497 | 33640/43456 |
| finn-b-F5_SCHIZO | Schizophrenia, schizotypal and delusional disorders | 6A20 Schizophrenia/6A22 Schizotypal disorder/MB26 Symptoms or signs involving content of thought | 16380466 | 10118/208674 |
| finn-b-G6_CERVROOT | Cervical root disorders | 8B9Y Other specified nerve root or plexus disorders | 16380406 | 131/195047 |
| finn-b-AUD_SWEDISH | Alcohol use disorder, Swedish definition | 6C40 Disorders due to use of alcohol | 16380466 | 12204/206588 |
| finn-b-F5_ALCOHOL_DEPENDENCE | Alcohol dependence | [6C40 Disorders due to use of alcohol](https://icd.who.int/browse11/l-m/en#/http%3a%2f%2fid.who.int%2ficd%2fentity%2f1676588433) | 16380440 | 5876/205659 |
| finn-b-F5_ALCOHOL | Mental and behavioural disorders due to alcohol | 6C40 Disorders due to use of alcohol | 16380460 | 11100/205659 |
| finn-b-ALCOPOLYNEU | Alcohol induced polyneuropathy (ALCOPOLYNEU) | 8D44 Alcohol-related neurological disorders | 16380466 | 125/218667 |
| ukb-d-20544_6 | Mental health problems ever diagnosed by a professional: Panic attacks | 6B03 Specific phobia | 12509516 | 6518/111204 |
| finn-b-G6_BENINTRAHYP | Benign intracranial hypertension | 8D60 Increased intracranial pressure | 16380402 | 102/205799 |
| finn-b-CD2_BENIGN_MENINGES_EXALLC | Benign neoplasm of meninges (all cancers excluded) | 2A01 Primary neoplasms of meninges (Neoplasms of brain or central nervous system) | 16380339 | 1280/180320 |
| finn-b-CD2_BENIGN_MENINGES_SPINAL | Benign neoplasm: Spinal meninges | 2A01 Primary neoplasms of meninges | 16380466 | 118/218674 |
| finn-b-CD2_BENIGN_SPINAL_CORD | Benign neoplasm: Spinal cord | 2A02 Primary neoplasm of spinal cord, cranial nerves or remaining parts of central nervous system | 16380466 | 196/218596 |
| finn-b-CD2_BENIGN_CRANIAL_NERVE | Benign neoplasm: Cranial nerves | 2A02 Primary neoplasm of spinal cord, cranial nerves or remaining parts of central nervous system | 16380466 | 357/218435 |
| finn-b-CD2_BENIGN_BRAIN_SUPRATENT_EXALLC | Benign neoplasm: Brain, supratentorial (all cancers excluded) | 2A00 Primary neoplasms of brain (Neoplasms of brain or central nervous system) | 16380337 | 218/180657 |
| finn-b-CD2_BENIGN_BRAIN_NOS_EXALLC | Benign neoplasm: Brain, unspecified (all cancers excluded) | 2A00 Primary neoplasms of brain (Neoplasms of brain or central nervous system) | 16380337 | 87/180717 |
| finn-b-CD2_BENIGN_PERIPH_AUTO_NEURO | Benign neoplasm: Peripheral nerves and autonomic nervous system | 2F3Y Benign non-mesenchymal neoplasms of other specified site | 16380466 | 204/218588 |
| finn-b-I9_INTRACRA | Nontraumatic intracranial haemmorrhage | 8B0Z Intracranial haemorrhage, unspecified | 16380408 | 2794/203068 |
| finn-b-I9_THROMBICV | Nonpyogenic thrombosis of intracranial venous system | 8B22 Certain specified cerebrovascular diseases | 16380404 | 108/203068 |
| finn-b-G6_CAUDA | Cauda equina syndrome | 8B40 Cauda equina syndrome | 16380461 | 149/216992 |
| ukb-b-8961 | Non-cancer illness code, self-reported: chronic fatigue syndrome | 8E49 Postviral fatigue syndrome | 9851867 | 2076/460857 |
| finn-b-G6_ENCEPATH | Encephalopathy | 8E47 Encephalopathy, not elsewhere classified | 16380402 | 110/205799 |
| finn-b-G6_ALCODEGEN | Degeneration of the brain due to alcohol | 8D44 Alcohol-related neurological disorders | 16380462 | 313/214239 |
| finn-b-I9_CERATHER | Cerebral atherosclerosis (I9_CERATHER) | BD55 Asymptomatic stenosis of intracranial or extracranial artery | 16380402 | 104/203068 |
| finn-b-C3_BRAIN | Malignant neoplasm of brain | 2A00 Primary neoplasms of brain | 16380466 | 464/218328 |
| ukb-d-I63 | Diagnoses - main ICD10: I63 Cerebral infarction | 8B11 Cerebral ischaemic stroke | 10889323 | 2353/358841 |
| finn-b-G6_HYDROCEPH | Hydrocephalus | 8D64 Hydrocephalus | 16380404 | 749/205799 |
| finn-b-G6_HCCOMM | Communicating hydrocephalus | 8D64 Hydrocephalus | 16380465 | 212/218043 |
| finn-b-C3_GBM | Brain glioblastoma | 2A00 Primary neoplasms of brain | 16380466 | 91/218701 |
| finn-b-C3_MENINGES_EXALLC | Malignant neoplasm of meninges (all cancers excluded) | 2A01 Primary neoplasms of meninges | 16380305 | 640/174006 |
| finn-b-G6_CERCYST | Cerebral cysts | 8E4Y Other specified disorders of the nervous system | 16380425 | 740/205799 |
| finn-b-G6_TIA | Transient ischemic attack (G6_TIA) | 8B10 Transient ischaemic attack | 16380437 | 8835/205799 |
| finn-b-G6_ANOXBRAINDAM | Anoxic brain damage | 8E65 Anoxic-ischaemic encephalopathy | 16380425 | 191/205799 |
| finn-b-G6_CPETAL | Cerebral palsy and other paralytic syndromes | 8D2Z Cerebral palsy, unspecified/MB5Z Paralytic symptoms, unspecified | 16380466 | 1800/216992 |
| finn-b-I9_VASCSYND | Vascular syndromes of brain in cerebrovascular disorders (I9_VASCSYND) | 8B26 Vascular syndromes of brain in cerebrovascular diseases | 16380453 | 591/210801 |
| finn-b-I9_CEREBVASC | Cerebrovascular diseases | Cerebrovascular diseases | 16380466 | 15724/203068 |
| ieu-b-7 | Parkinson's disease | 8A00 Parkinsonism | 17891936 | 33674/449056 |
| finn-b-PD2ND | Secondary parkinsonism | 8A00 Parkinsonism | 16380459 | 417/216346 |
| ukb-b-16868 | Non-cancer illness code, self-reported: migraine | 8A80 Migraine | 9851867 | 13597/449336 |
| finn-b-G6_MIGRAINE_WITH_AURA | Migraine with aura | 8A80 Migraine | 16380353 | 3541/176107 |
| finn-b-MIGRAINE_TRIPTAN | Migraine, single triptan purchase ok & required. ICD-code if available is included | 8A80 Migraine | 16380466 | 19676/199116 |
| finn-b-F5_PARAPER | Paranoid personality disorder | 6D10 Personality disorder | 16380428 | 354/212179 |
| finn-b-G6_TRIOTHUNS | Other and unspecified trigeminal disorders | 8B82 Disorders of trigeminal nerve | 16380408 | 221/195047 |
| ieu-a-1189 | Obsessive Compulsive Disorder | 6B2Z Obsessive-compulsive or related disorders, unspecified | 8409517 | 26888/7037 |
| ukb-a-528 | Diagnoses - main ICD10: G56 Mononeuropathies of upper limb | 8C10 Mononeuropathies of upper limb | 10894596 | 5886/331313 |
| finn-b-G6_BRACHPLE | Brachial plexus disorders | 8B91 Brachial plexus disorders | 16380407 | 1283/195047 |
| finn-b-G6_ULLNLE | Lesion of ulnar nerve | 8C10 Mononeuropathies of upper limb | 16380416 | 2006/195047 |
| finn-b-G6_FEMLE | Lesion of femoral nerve | 8C11 Mononeuropathies of lower limb | 16380406 | 101/195047 |
| finn-b-G6_FEMLAPOP | Lesion of lateral popliteal nerve | 8C11 Mononeuropathies of lower limb | 16380406 | 624/195047 |
| finn-b-G6_ICNEUROP | Intercostal neuropathy | 8C12 Certain specified mononeuropathies | 16380406 | 91/195047 |
| finn-b-G6_RADLES | Lesion of radial nerve | 8C10 Mononeuropathies of upper limb | 16380410 | 534/195047 |
| finn-b-G6_TRINEU | Trigeminal neuralgia | 8B82 Disorders of trigeminal nerve | 16380408 | 800/195047 |
| ukb-d-G57 | Diagnoses - main ICD10: G57 Mononeuropathies of lower limb | 8C11 Mononeuropathies of lower limb | 9584367 | 1111/360083 |
| finn-b-G6_LSROOT | Lumbosacral root disorders, not elsewhere classified | 8B9Y Other specified nerve root or plexus disorders | 16380414 | 232/195047 |
| finn-b-G6_HEREMOSEN | Hereditary monor and sensory neuropathy | 8C20 Hereditary motor and sensory neuropathy | 16380463 | 98/215718 |
| finn-b-ALCONEURODEGEN | Degeneration of nervous system due to alcohol | 8D44 Alcohol-related neurological disorders | 16380466 | 313/218479 |
| finn-b-G6_PLANTAR | Lesion of plantar nerve | 8C11 Mononeuropathies of lower limb | 16380407 | 1017/195047 |
| finn-b-G6_SCIALE | Lesion of sciatic nerve | 8C11 Mononeuropathies of lower limb | 16380407 | 781/195047 |
| finn-b-M13_RADICULOPATHY | Radiculopathy | 8B93 Radiculopathy | 16380228 | 2886/164682 |
| ieu-a-816 | Neuroblastoma | XH85Z0 Neuroblastoma, NOS (Neuroepitheliomatous neoplasms) | 468788 | 1627/3254 |
| finn-b-F5_BULIMIA | Bulimia nervosa (incl. atypical) | 6B81 Bulimia Nervosa | 16380456 | 547/213826 |
| ieu-a-1186 | Anorexia Nervosa | 6B80 Anorexia Nervosa | 10641224 | 3495/10982 |
| finn-b-F5_INSOMNIA | Insomnia | 7A0Z Insomnia disorders, unspecified | 16380465 | 1691/216164 |
| finn-b-AMN2 | Transient global amnesia | MB21 Symptoms, signs or clinical findings involving cognition (Mental or behavioural symptoms, signs or clinical findings) | 16380453 | 1230/210148 |
| finn-b-F5_SUBSTANCE | Mental and behavioural disorders due to psychoctive substance use | 6C4G Disorders due to use of unknown or unspecified psychoactive substances | NA | 13133/205659 |
| finn-b-H7_OPTNERVE | Disorders of optic nerve and visual pathways | 9C40 Disorder of the optic nerve | 16380466 | 1301/217491 |
| finn-b-H7_OPTNEURITIS | Optic neuritis | 9C40 Disorder of the optic nerve | 16380463 | 582/217491 |
| ieu-b-41 | bipolar disorder | 6A6Z Bipolar or related disorders, unspecified | 13413244 | 20352/31358 |
| ukb-d-20126_0 | Bipolar and major depression status: No Bipolar or Depression | 待分类mental | 13562962 | 62825/24070 |
| finn-b-G6_NARCOCATA | Narcolepsy and cataplexy | 7A20 Narcolepsy | 16380329 | 88/176107 |
| finn-b-F5_HYPERSOMNIA | Hypersomnia | 7A21 Idiopathic hypersomnia | 16380458 | 176/216164 |
| ebi-a-GCST005522 | Narcolepsy | 7A20 Narcolepsy | 93613 | 1886/10421 |
| ukb-b-16781 | Diagnoses - main ICD10: G47.3 Sleep apnoea | 7A4Z Sleep-related breathing disorders, unspecified | 9851867 | 2320/460690 |
| finn-b-F5_SLEEPWAKE | Disorder of the sleep-wake schedule | 7A6Z Circadian rhythm sleep-wake disorders, unspecified | 16380459 | 190/216164 |
| finn-b-SLEEP | Sleep disorders (combined) | 7B2Z Sleep-wake disorders, unspecified | 16380458 | 19155/197545 |
| finn-b-DM_POLYNEURO | Diabetic polyneuropathy | 8C03 Other secondary polyneuropathy | 16380465 | 358/217377 |
| finn-b-G6_DIABETNEUR | Diabethic neuropathy | 8C03 Other secondary polyneuropathy | 16380416 | 1419/195047 |
| finn-b-G6_HEADACHE | Other headache syndromes | 8A8Z Headache disorders, unspecified | 16380359 | 7952/176107 |
| finn-b-R18_HEADACHE1 | Headache | 8A8Z Headache disorders, unspecified | 16380371 | 13345/172999 |
| ukb-b-13092 | Headaches for 3+ months | 8A8Z Headache disorders, unspecified | 9851867 | 41719/49550 |
| finn-b-G6_CARPTU | Carpal tunnel syndrome | 8C10 Mononeuropathies of upper limb | 16380439 | 11208/195047 |
| ukb-b-3965 | Diagnoses - main ICD10: G56.0 Carpal tunnel syndrome | 8C10 Mononeuropathies of upper limb | 9851867 | 8289/454721 |
| finn-b-F5_DELUSIO | Persistent delusional disorders | 6A24 Delusional disorder | 16380426 | 1665/208674 |
| finn-b-I9_ANEURYSM | Cerebral aneurysm, nonruptured | 8B22 Certain specified cerebrovascular diseases | 16380404 | 992/203068 |
| finn-b-SMOKING_DEPEND | Smoking dependancy | 6C4A Disorders due to use of nicotine | 16380466 | 962/217471 |
| finn-b-F5_HABIT | Habit and impulse disorders | 6C7Y Other specified impulse control disorders | 16380431 | 260/212179 |
| finn-b-CHARCOT | Charcot foot | FA38.1Z Neuropathic arthropathy, unspecified | 16380187 | 137/162201 |
| finn-b-G6_ATROPHCIRCUM | Circumscribed brain atrophy | 8E4Y Other specified disorders of the nervous system | 16380462 | 235/214239 |
| finn-b-SFN | Small fibre neuropathy | 8C0Y Other specified polyneuropathy | 16380463 | 243/215718 |
| finn-b-F5_GENDER | Gender identity disorders | HA61 Gender incongruence of childhood | 16380428 | 137/212179 |
| finn-b-KRA_PSY_ALCOH | Alcohol abuse, main dg | [6C40 Disorders due to use of alcohol](https://icd.who.int/browse11/l-m/en#/http%3a%2f%2fid.who.int%2ficd%2fentity%2f1676588433) | 16380466 | 2660/216132 |
| finn-b-KRA_PSY_SPEELING | Speech and linguistic disorders | 6A01 Developmental speech or language disorders | 16380466 | 1312/217480 |
| finn-b-G6_DRUGPOLY | Drug-induced polyneuropathy | 8D43 Neurological disorders due to toxicity | 16380463 | 93/215718 |
| finn-b-G6_HERATAXIA | Hereditary ataxia | 8A03 Ataxic disorders | 16380466 | 154/218269 |
| finn-b-F5_DEPRESSIO | Depression | 6A7Z Depressive disorders, unspecified | 16380457 | 23424/192220 |
| ieu-b-102 | Major depression | 6A70 Single episode depressive disorder | NA | 170756/329443 |
| ukb-b-12064 | Non-cancer illness code, self-reported: depression | 6A7Z Depressive disorders, unspecified | 9851867 | 26595/436338 |
| ebi-a-GCST005902 | Depression (broad) | 6A7Z Depressive disorders, unspecified | 7624934 | 113769/208811 |
| finn-b-O15_POSTPART_DEPR | Postpartum depression | 6E20 Mental or behavioural disorders associated with pregnancy, childbirth or the puerperium, without psychotic symptoms | 16376275 | 7604/59601 |
| finn-b-F5_EATING | Eating disorders | 6B8Z Feeding or eating disorders, unspecified | 16380466 | 1874/216918 |
| ukb-b-11542 | Manic/hyper symptoms: I was more active than usual | 6A60 Bipolar type I disorder | 9851867 | 7877/23440 |
| ukb-b-8893 | Manic/hyper symptoms: I was more talkative than usual | 6A60 Bipolar type I disorder | 9851867 | 6523/24794 |
| ukb-b-13187 | Manic/hyper symptoms: I was more creative or had more ideas than usual | 6A60 Bipolar type I disorder | 9851867 | 3745/27572 |
| ukb-b-11588 | Manic/hyper symptoms: I needed less sleep than usual | 6A60 Bipolar type I disorder | 9851867 | 4245/27072 |
| ukb-d-20548_2 | Manifestations of mania or irritability: I was more restless than usual | 6A60 Bipolar type I disorder | 13571113 | 12876/101546 |
| ukb-d-20548_6 | Manifestations of mania or irritability: I was more creative or had more ideas than usual | 6A60 Bipolar type I disorder | 11200219 | 3090/111332 |
| ukb-d-20548_8 | Manifestations of mania or irritability: I was more confident than usual | 6A60 Bipolar type I disorder | 11306770 | 3285/111137 |
| ukb-d-20548_3 | Manifestations of mania or irritability: My thoughts were racing | 6A60 Bipolar type I disorder | 13364555 | 10580/103842 |
| ukb-d-20548_7 | Manifestations of mania or irritability: I was easily distracted | 6A60 Bipolar type I disorder | 13113620 | 9162/105260 |
| finn-b-F5_DELIRIUM | Delirium, not induced by alcohol and other psychoactive substances | 6D70 Delirium | 16380452 | 1269/209487 |
| finn-b-F5_ANAPER | Anankastic personality disorder | 6D10 Personality disorder | 16380429 | 476/212179 |
| finn-b-KRA_PSY_HYPERKIN | Hyperkinetic disorders | 6A05 Attention deficit hyperactivity disorder | 16380466 | 955/217837 |
| ieu-a-1183 | ADHD | 6A05 Attention deficit hyperactivity disorder | 8047420 | 20183/35191 |
| finn-b-F5_MIXPER | Mixed and other personality disorders | 6D10 Personality disorder | 16380434 | 1666/212179 |
| finn-b-F5_PSYTRANS | Acute and transient psychotic disorders | 6A23 Acute and transient psychotic disorder | 16380441 | 3281/208674 |
| finn-b-F5_ANXPER | Anxious personality disorder | 6D10 Personality disorder | 16380428 | 280/212179 |
| finn-b-F5_OPIOIDS | Mental and behavioural disorders due to opioids | 6C43 Disorders due to use of opioids | 16380458 | 651/214999 |
| finn-b-KRA_PSY_CHILDEMOT | Emotional disorders starting during childhood or adolecense | Mental and emotional disorders | 16380466 | 805/217987 |
| finn-b-F5_EMOPER | Emotionally unstable personality disorder | 6D10 Personality disorder | 16380456 | 2637/212179 |
| finn-b-F5_MOOD | Mood [affective] disorders | 6A8Z Mood disorders, unspecified | 16380466 | 26572/192220 |
| finn-b-KRA_PSY_PERSON | Personality disorders | 6D10 Personality disorder | 16380466 | 6254/212538 |
| finn-b-F5_DISPER | Dissocial personality disorder | 6D10 Personality disorder | 16380430 | 340/212179 |
| finn-b-F5_DEPPER | Dependent personality disorder | 6D10 Personality disorder | 16380428 | 411/212179 |
| finn-b-F5_PERSONALITY | Specific personality disorders | 6D10 Personality disorder | 16380464 | 5409/212179 |
| finn-b-KRA_PSY_CODUCTEMOT | Mixed disorders of conduct and emotions (KRA_PSY_CODUCTEMOT) | 6C91 Conduct-dissocial disorder | 16380466 | 416/218376 |
| finn-b-KRA_PSY_BEHAV | Behavioural disorders | 6E8Z Mental, behavioural or neurodevelopmental disorders, unspecified | 16380466 | 400/218392 |
| finn-b-F5_PERSOBEH | Disorders of adult personality and behaviour | 6E68 Secondary personality change | 16380466 | 6613/212179 |
| finn-b-F5_CANNABIS | Mental and behavioural disorders due to cannabinoids | 6C42 Disorders due to use of synthetic cannabinoids | 16380457 | 617/214999 |
| finn-b-KRA_PSY_CHILDSOC_EXMORE | Social disorders starting during childhood or adolecense (more controls excluded) | 6C91 Conduct-dissocial disorder | 16380155 | 129/166584 |
| finn-b-F5_TOBAC | Mental and behavioural disorders due to tobacco | 6C4A Disorders due to use of nicotine | 16380434 | 962/214999 |
| finn-b-F5_STIMUL | Mental and behavioural disorders due to use of other stimulants, including caffeine | 6C48 Disorders due to use of caffeine | 16380435 | 571/214999 |
| finn-b-F5_SEDAHYP | Mental and behavioural disorders due to sedatives or hypnotics | 6C44 Disorders due to use of sedatives, hypnotics or anxiolytics | 16380462 | 1255/214999 |
| finn-b-G6_XTRAPYR | Extrapyramidal and movement disorders | 8A0Z Movement disorders, unspecified | 16380466 | 4948/213844 |
| finn-b-F5_MENTRET | Mental retardation (F5_MENTRET) | 6A00 Disorders of intellectual development | 16380466 | 761/218031 |
| finn-b-F5_MODRET | Moderate mental retardation | 6A00 Disorders of intellectual development | 16380466 | 104/218031 |
| ukb-b-8714 | Vascular/heart problems diagnosed by doctor: Stroke | 8B11 Cerebral ischaemic stroke | 9851867 | 7055/454825 |
| finn-b-I9_STR_SAH | Stroke, including SAH | 8B20 Stroke not known if ischaemic or haemorrhagic | 16380445 | 12632/200315 |
| ebi-a-GCST006906 | Stroke | 8B20 Stroke not known if ischaemic or haemorrhagic | 8211693 | 40585/406111 |
| ebi-a-GCST006908 | Ischemic stroke | 8B11 Cerebral ischaemic stroke | 8296492 | 34217/406111 |
| ebi-a-GCST006907 | Ischemic stroke (large artery atherosclerosis) | 8B11 Cerebral ischaemic stroke | 8418349 | 4373/406111 |
| ebi-a-GCST006909 | Ischemic stroke (small-vessel) | 8B11 Cerebral ischaemic stroke | 8280845 | 5386/192662 |
| ebi-a-GCST005842 | Ischemic stroke (cardioembolic) | 8B11 Cerebral ischaemic stroke | 7954834 | 7193/406111 |
| ebi-a-GCST006910 | Ischemic stroke (cardioembolic) | 8B11 Cerebral ischaemic stroke | 8271294 | 7193/406111 |
| ieu-a-1109 | Cardioembolic stroke | 8B11 Cerebral ischaemic stroke | 2421920 | 1859/19326 |
| finn-b-G6_MYASTHENIA | Myasthenia gravis | 8C60 Myasthenia gravis | NA | 232/217056 |
| finn-b-I9_SAH | Subarachnoid haemmorrhage | 8B01 Subarachnoid haemorrhage | 16380395 | 1338/201230 |
| ukb-b-1220 | Diagnoses - secondary ICD10: G55.1 Nerve root and plexus compressions in intervertebral disk disorders | 8B90 Nerve root and plexus compressions | 9851867 | 2298/460712 |
| ieu-a-1185 | Autism Spectrum Disorder | 6A02 Autism spectrum disorder | 9112386 | 18382/27969 |

**Abbreviations:** GWAS Dataset ID, Genome wide association study summary dataset ID; Tait, Neurological disorders; ICD-11 codes, Neurological diseases are classified in the ICD-11 under the specific category for the nervous system; Number of SNPs, The total number of Single Nucleotide Polymorphisms in the neurological diseases; Sample size (case/control), The number of individuals in the case and control groups

**Figure S1** Neighborhood size plot

**Figure S2** Pathway analysis of fGSEA revealed significantly regulated curated gene sets pathways in astrocyte, 10 top and bottom regulated pathways are shown.

**Figure S3** Pathway analysis of fGSEA revealed significantly regulated Gene Ontology (GO) pathways in astrocyte, 10 top and bottom regulated pathways are shown.

**Figure S4** Pathway analysis of fGSEA revealed significantly regulated curated gene sets pathways in oligodendrocyte, 10 top and bottom regulated pathways are shown.

**Figure S5** Pathway analysis of fGSEA revealed significantly regulated Gene Ontology (GO) pathways in oligodendrocyte, 10 top and bottom regulated pathways are shown.
